# MicroRNA-143/-145 in Cardiovascular Diseases

**DOI:** 10.1155/2015/531740

**Published:** 2015-06-28

**Authors:** Wang Zhao, Shui-Ping Zhao, Yu-Hong Zhao

**Affiliations:** Department of Cardiology, The Second Xiangya Hospital of Central South University, No. 139, People Street, Changsha, Hunan 410011, China

## Abstract

MicroRNAs (miRNAs) play an essential role in the onset and development of many cardiovascular diseases. Increasing evidence shows that miRNAs can be used as potential diagnostic biomarkers for cardiovascular diseases, and miRNA-based therapy may be a promising therapy for the treatment of cardiovascular diseases. The microRNA-143/-145 (miR-143/-145) cluster is essential for differentiation of vascular smooth muscle cells (VSMCs) and determines VSMC phenotypic switching. In this review, we summarize the recent progress in knowledge concerning the function of miR-143/-145 in the cardiovascular system and their role in cardiovascular diseases. We discuss the potential role of miR-143/-145 as valuable biomarkers for cardiovascular diseases and explore the potential strategy of targeting miR-143 and miR-145.

## 1. Introduction

MicroRNAs (miRNAs) are short 18–24 nucleotide, single-stranded, noncoding RNAs that bind to the complementary target sites in 3′-untranslated regions (3′-UTRs) of specific mRNA targets to inhibit translation or to cause mRNA degradation [1]. It is estimated that the human genome contains more than 1,000 miRNAs, which regulate at least 30% of protein-coding genes [2–4]. In addition, one single miRNA can exert inhibitory effects on many mRNAs, whereas one single mRNA can be modulated by many miRNAs. Thus, the regulation of the expression of mRNAs by miRNAs is a complex process that involves diverse cellular functions: cell proliferation and differentiation, apoptosis, neuronal patterning, immunity, fat metabolism, and phenotypic switching of vascular smooth muscle cells (VSMCs) [5, 6]. Dysregulation of several miRNAs is associated with many diseases including cancer, cardiovascular diseases, and neurological disorders [7–10]. MiRNA-based therapy has become a promising treatment for many human diseases including cardiovascular diseases and cancer [11–13].

Cardiovascular diseases are the leading cause of morbidity and mortality worldwide. Although it has been well established that genetic mutations and cellular mechanisms contribute to the pathogenesis of several cardiovascular diseases, recent studies have shown that miRNAs play key roles in cardiovascular system development [4, 14–16]. It has been determined that miRNAs contribute to many cardiovascular processes, such as embryonic stem cell differentiation, cardiomyocyte proliferation, VSMC phenotypic switching, endothelial responses to shear stress, and erythropoiesis [4, 6, 17–21]. Dysregulation of miRNAs has been found in many cardiovascular diseases including heart failure, myocardial ischemia, congenital heart disease, atherosclerosis, and hypertension [22–27].

The microRNA-143/-145 (miR-143 and miR-145) encoding genes are located in close proximity with each other on human chromosome 5 and are believed to be cotranscribed in the same bicistronic transcript [28]. The miR-143/-145 gene cluster is expressed in the heart and in VSMCs [29–32]. MiR-143 is believed to play an essential role in the function and formation of the cardiac chamber via regulation of myocardial cell morphology [32]. MiR-143 and miR-145 are essential for VSMC differentiation [31] and are molecular keys to determine VSMC phenotypic switching [6, 30]. It is interesting that miR-143 and miR-145 can be upregulated in endothelial cells in response to shear stress and subsequently are exported in exosome-like vesicles that regulate VSMC phenotype [33]. In addition, it has been reported that circulating miR-145 levels differ in patients with coronary artery disease [34, 35]; in patients with acute myocardial infarction (AMI), the level of miR-145 in total peripheral blood correlates with infarct size [36]. This suggests that miR-145 could be a valuable biomarker for cardiovascular diseases. Furthermore, several clinical studies have shown that miR-143/-145 dysregulation is associated with many cardiovascular diseases, including essential hypertension, atherosclerosis, pulmonary arterial hypertension, and coronary artery disease [35, 37–40]. Therefore, targeting miR-143/-145 may be a promising therapeutic strategy for the treatment of these cardiovascular diseases [41].

In this review, we describe the function of miR-143 and miR-145 in the cardiovascular system and discuss their roles in cardiovascular diseases. In addition, we explore whether miR-143 and miR-145 may be valuable biomarkers for cardiovascular diseases and whether targeting miR-143/-145 could be a potential strategy for the treatment of cardiovascular diseases.

## 2. The Functions of MiR-143 and MiR-145 in the Cardiovascular System

### 2.1. VSMCs

**Figure 1:**
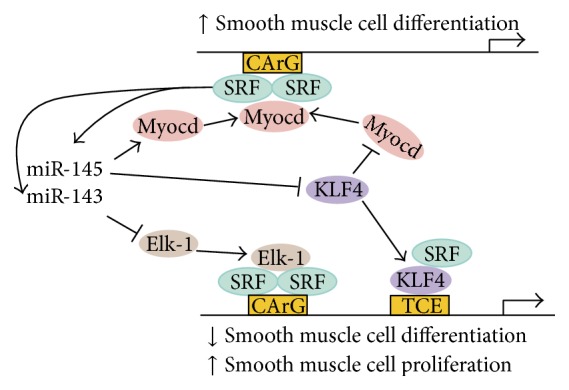
Roles of miR-143/-145 in the regulation of vascular smooth muscle cell differentiation and proliferation (adapted from Cordes et al. [31]). Expressions of miR-143/-145 are positively regulated by SRF with the functions to repress several factors, including KLF4 and Elk-1, which are reported to be involved in the regulation of smooth muscle cell proliferation, leading to promotion of smooth muscle cell differentiation and inhibition of proliferation. SRF: serum response factor; Myocd: myocardin; KLF4: Kruppel-like factor 4; Elk-1: Ets-like gene 1.

MiR-143 and miR-145 are abundantly expressed in VSMCs [29–31, 43, 44]. Both miR-143 and miR-145 play crucial roles in VSMC differentiation [29, 31, 43]. Overexpression of miR-145 upregulates the expression of VSMC differentiation marker genes, such as smooth muscle alpha-actin, calponin, and smooth muscle-myosin heavy chain (SM-MHC), thus promoting differentiation of VSMCs into the contractile phenotype [30]. The effect of miR-145 on VSMC phenotypic modulation is through the suppression of Kruppel-like factor 5 (KLF5) and Kruppel-like factor 4 (KLF4) and subsequent stimulation of their downstream signaling molecule myocardin [30, 45] (Figure 1). Furthermore, miR-145 has been found to be sufficient to induce differentiation of multipotent neural crest stem cells into VSMCs [31]. Similarly, miR-143 can regulate VSMC phenotype similar to miR-145, but to a lesser extent [31]. In addition to KLF5, KLF4, and myocardin, many other targets of miR-143/-145 have been identified, including Slit-Robo GTPase-activating protein 1 (Srgap1), Srgap2, Adducin-3, Slingshot 2 (Ssh2), angiotensin converting enzyme (ACE), calmodulin kinase II *δ*, fascin, and myocardin-related transcription factor-*β* (MRTF-*β*) [29–31, 42, 46] (Table 1). Moreover, the expression of miR-143/-145 is induced by the serum response factor (SRF) and its coactivator myocardin and regulated by cytoskeletal dynamics and responses of VSMCs to injury. This suggests that they are an integral component of a complex regulatory network of SRF in the control of VSMC phenotypic switching [42].

The role of miR-143/-145 in VSMC was further investigated by studies in knockout mice. MiR-143/-145 knockout mice exhibit many abnormalities in VSMCs, including a reduction in marker expression, decreased contractile function, increased and dilated rough endoplasmic reticulum, thin smooth muscle layer, and decreased actin-based stress fibers [29, 42, 43]. These structural functional findings indicate that miR-143/-145 knockout results in a shift from a contractile to synthetic phenotype of VSMCs, suggesting that miR-143 and miR-145 are required for maintaining the contractile phenotype and function of VSMCs. Although miR-143/-145 play an important role in determining VSMC phenotype and function, it appears that they are not essential for cardiovascular development* in vivo*, since miR-143/-145 knockout mice are viable and do not have gross macroscopic alterations [29, 42, 43]. This is consistent with the finding that miR-143 is only active during the early stages of heart development and disappears from cardiomyocytes at later stages [29]. In contrast, the Dicer-knockout mice are lethal by extensive internal hemorrhage at the later embryonic stage [47]. These findings suggest that other miRNAs, but not miRNA-143 and miRNA-145, are essential for VSMC development* in vivo*.

### 2.2. Endothelium

MiR-143 and miR-145 have been shown to be upregulated in endothelial cells in response to shear stress [33, 48]. In human aortic arterial endothelial cells, shear stress-induced upregulation of miR-145 decreases the expression of its target gene junctional adhesion molecule-A (JAM-A), a molecule that induces inflammatory cell entry into the atherosclerotic site [48]. MiR-143 and miR-145 have been known to control the expression of various proteins that are involved in the regulation of actin cytoskeleton [33, 42, 49]. Although it remains unclear whether miR-143 and miR-145 contribute to actin cytoskeleton rearrangement induced by shear stress in endothelial cells [50], miR-143 and miR-145 have been found to be upregulated in endothelial cells in response to KLF2 and shear stress [33]. MiR-143/-145 are released from endothelial cells in exosome-like vesicles and subsequently regulate target gene expression in VSMCs [33]. It appears that miR-143/-145 may function as a signaling molecule that communicates between endothelial cells and VSMCs.

### 2.3. Heart

MiR-143/-145 have been found to be expressed in the heart [31, 32, 51]. MiR-143 controls F-actin dynamics and cell morphology and is essential for cardiac chamber function and morphogenesis [32]. MiR-143 expression is found to be regulated by heart beat [52]. In addition, the expression of miR-143/-145 is restricted to an early stage of heart development and disappears from cardiomyocytes at E16.5 [29, 42]. MiR-145 has been found upregulated in the heart of mice after transverse aortic constriction and inhibits isoproterenol-induced cardiomyocyte hypertrophy [51]. The expression of miR-143/-145 in cardiomyocytes can be induced by activin A via the p38 mitogen-activated protein kinase (MAPK) signaling pathway, resulting in inhibition on insulin-mediated biological action [53]. Furthermore, the expression level of miR-145 exhibited a tendency toward a higher expression in patients with end-stage dilated cardiomyopathy compared with healthy controls, though no significant difference was found [54].

### 2.4. MiR-143 and MiR-145 in the Plasma

MiRNAs are present in the peripheral blood cells such as erythrocytes and mononuclear cells [19, 55]. The miR-143/-145 complex has been found to be downregulated in M1 microphages compared with M2 macrophages, suggesting that miR-143/-145 may play an important role in macrophage differentiation and polarized activation processes [56]. In addition, microRNAs can be stably present in the serum, and thus circulating miRNAs can be used for potential biomarkers for many diseases (Table 2). MiR-143/-145 in human peripheral blood mononuclear cells have been found to be upregulated in patients with essential hypertension [38]. Changes in circulating miR-145 levels were found in patients with stable coronary artery disease [35], stable or unstable angina [34], and acute myocardial infarction [36]. Therefore, miR-143 and miR-145 can be used for potential biomarkers for these cardiovascular diseases.

## 3. Role of MiR-143/-145 in Atherosclerosis and Hypertension

### 3.1. Atherosclerosis

Atherosclerosis is a chronic disease of the vascular wall involving multiple pathological processes, such as lipid retention and inflammations in many different types of cells such as various inflammatory cells, endothelial cells, and VSMCs [57]. Since miR-143 and miR-145 are expressed in endothelial cells, VSMCs, and inflammatory cells, it is not surprising that several clinical and animal studies have shown that miR-143 and miR-145 contribute to pathogenesis of atherosclerosis [33, 39, 58–60]. The role of miR-143/-145 in atherosclerosis may result from their regulation of endothelial cells, VSMCs, and circulating blood cells.

VSMCs play a role in the pathogenesis of atherosclerosis [61]. The miR-145 cluster has been found to be downregulated in the proliferative VSMCs of atherosclerotic arteries in ApoE-knockout mice [58]. This suggests that downregulation of miR-145 may contribute to atherogenesis. Consistent with this idea, miR-143/-145 knockout mice with normal serum concentration of triglycerides and lipoproteins exhibited spontaneous atherosclerotic lesion in the femoral artery [29]. Furthermore, overexpression of miR-145 in VSMCs reduced plague size in aortic sinuses, ascending aortas, and brachiocephalic arteries in ApoE-knockout mice [59]; this further confirms the atheroprotective role of miR-145. However, the atheroprotective mechanisms of miR-143/-145 have not been well elucidated, even though many potential targets of miR-143/-145, such as ACE [29], are involved in the pathogenesis of atherosclerosis [62]. The atheroprotective role of miR-143 and miR-145 may be attributed to their ability to promote contractile VSMC phenotype and to inhibit the synthetic VSMC phenotype that is associated with atherosclerosis [29, 57]. In agreement with this hypothesis is that miRNAs, including miR-145 that are associated with the contractile VSMC phenotype, are downregulated and some miRNAs, such as miR-21, 146a and 221 that are connected to the synthetic VSMC phenotype, are upregulated in atherosclerotic plaques [63].

Endothelial cells are subject to different regimes of shear stress in distinct areas of blood vessels. Atherosclerotic lesions are susceptible to occurring in areas of disturbed flow and low shear stress, whereas unbranched portions of arteries with uniform laminar flow are protected from atherogenesis. It has been reported that miR-143/-145 are upregulated in human aortic arterial endothelial cells and human umbilical vein endothelial cells in response to atheroprotective laminar flow [33, 48]. The exact mechanisms underlying atheroprotective effect of miR-143/-145 remains unclear. Hergenreider et al. reported that KLF2 transduction and shear stress induced upregulation of miR-143/-145 in endothelial cells, which were released in exosome-like vesicles and inhibited the target gene expression in VSMCs [33]. Furthermore, extracellular vesicles derived from KLF2-expression endothelial cells reduced aortic atherosclerosis in ApoE-knockout mice [33], suggesting that miR-143/-145 may exert atheroprotective role via a route of communication between endothelial cells and VSMCs. In addition, Schmitt et al. found that upregulation of miR-145 repressed junctional adhesion molecule-A- (JAM-A-) induced entry of monocytes into the sites of atherosclerotic lesions, suggesting that miR-145 may inhibit atherosclerotic formation via downregulation of JAM-A in endothelial cells [48].

In clinical studies, miR-145 has been found to be overexpressed in carotid atherosclerotic plaques in patients with symptomatic stroke compared with plaques in patients with nonsymptomatic stoke [64]. Recently, Santovito et al. reported that miR-145 was upregulated in atherosclerotic plaques of hypertensive patients compared with control plaques of nonhypertensive patients [39], suggesting that hypertension may upregulate miR-145 expression in human atherosclerotic plaque. These clinical findings that miR-145 is upregulated in atherosclerotic plaques are apparently in contrast with the animal studies showing that upregulation of miR-145 is atheroprotective [59]. This discrepancy may be explained by compensatory upregulation of miR-145 in response to chronic stress in patients with chronic diseases.

### 3.2. Hypertension

VSMCs determine vascular tone and regulate vascular resistances and thus play a fundament role in the development of hypertension [65]. The transition of VSMCs from a state of differentiation to a state of proliferation contributes to the pathogenesis of hypertension [66]. MiR-143/-145 knockout mice have a reduced blood pressure due to reduced vascular tone [29, 42, 67], suggesting that miR-143/-145 are required for controlling blood pressure.

MiR-143 and miR-145 have been found to be downregulated in VSMCs in response to acute and chronic vascular stress [30, 43]. In hypertension, VSMCs are constantly exposed to excessive stretch due to persisting high blood pressure. It has been reported that miR-145 is important for the expression of the VSMC contractile phenotype, [29, 31, 43] as it mediates stretch-induced differentiation of VSMCs [68, 69]. Recently, Hu et al. found that stretch-induced activation of extracellular signal-regulated kinase 1/2 (ERK1/2) and upregulation of ACE contribute to reduced expression of miR-145 because of a reduction in mechanical stretch [68]. Stretch-induced downregulation of miR-145 resulted in reduced expression of VSMCs contractile markers, including KLR4 and myocardin [68]. Therefore, modulation of the VSMC phenotype by miR-145 in response to persistent hypertensive stretch may contribute to the pathogenesis of hypertension.

Many of potential targets of miR-143/-145, such as CAMK-II and ACE, are involved in blood pressure regulation [29, 69]. MiR-145 can increase the expression of L-type calcium channel via suppression of CAMK-II [69]. L-type calcium channels that couple blood pressure-induced membrane potential change to the myogenic response in VSMCs has been known to contribute to the pathogenesis of hypertension [70]. In addition, ACE, a regulator of blood pressure, is upregulated in VSMCs of miR-145 knockout mice, resulting in an increased generation of angiotensin II in blood vessel walls [29]. ACE in VSMCs is known to play an important role in vascular remodeling in hypertension [71]. Therefore, many signaling pathways that are targeted by miR-145 may underlie the miR-145-mediated pathogenesis of hypertension.

Recently, Kontaraki et al. found that the expression levels of miR-143/-145 in human peripheral blood mononuclear cells were lower in hypertensive patients than in healthy controls and the expression levels negatively correlated with 24 hr diastolic and mean blood pressure [38]. This clinical finding that miR-143/-145 is downregulated in hypertensive patients is consistent with animal studies showing that the expression of miR-143/-145 is decreased in vascular walls after balloon carotid artery injury and in ApoE-knockout mice [30, 43, 58]. It is possible that a fraction of the miR-145 levels in human peripheral blood mononuclear cells may be transferred from the vascular wall.

Many microRNAs, including miR-145, have an important role in pathogenesis of pulmonary arterial hypertension (PAH) [41, 72]. MiR-145 has been shown to be activated by transforming growth factor-*β* (TGF-*β*) signaling pathway in VSMCs [73, 74] and upregulation of miR-145 by TGF-*β* to promote differentiation in lung myofibroblasts [75]. Caruso et al. reported that miR-145 is upregulated in PAH patients and in a mouse model of hypoxia-induced PAH, as well as in the lungs of mice with mutant bone morphogenetic protein (BMP) receptor type-2 (BMPR2), a receptor for the TGF-*β* superfamily [37]. Furthermore, miR-145 knockout results in a significant protection from the development of PAH [37], suggesting that miR-145 plays an important role in the development of PAH.

## 4. MiR-143/-145 as Potential Diagnostic Markers for Cardiovascular Diseases

In addition to control cellular processes, miRNAs are released into the circulation and can be stably present in the plasma [76]. Since miRNAs can be detected in the plasma under diseased conditions, circulating miRNAs may function as potential diagnostic markers for cardiovascular diseases [55, 76, 77]. Increasing evidence has shown that circulating miRNAs may function as diagnostic markers for coronary artery disease (CAD), diabetic heart diseases, myocardial infarction, hypertension, and heart failure [34, 55, 76, 78, 79].

Considering the specificity of microRNAs, which are short, single-strand, and noncoded, their measurement would be different from other coded genes. Currently there are three primary methods for detection and quantification of microRNAs, Northern blot, PCR, and microarray. Among them, PCR is the mostly used approach for microRNAs studies especially in clinical studies, due to high sensitivity and specificity as well as the ability to quantify the number of microRNAs present in each sample. Through using quantitative PCR approach, several studies have investigated the circulating miR-145 levels in patients with coronary artery disease [34, 35]. Fichtlscherer et al. reported that circulating miR-145 was downregulated in patients with stable coronary artery disease compared with healthy controls [35]. However, D'Alessandra et al. found that circulating miR-145 was upregulated in patients with unstable angina compared with healthy controls [34]. This discrepancy may be associated with different disease status (stable CAD by Fichtlscherer et al. versus unstable angina by D'Alessandra et al.). More miR-145 may be released from vascular walls due to more severe injuries in patients with unstable angina compared with patients with stable CAD. This hypothesis agrees with the findings that plasma concentrations of miR-145 correlate with muscle injury in rodents [44]. In addition, both studies used a limited number of patients (19 patients with unstable angina versus 20 controls in D'Alessandra's study and 36 patients with CAD versus 17 controls in Fichtlscherer's study). Further studies with a large sample size are required to demonstrate the role of miR-145 as a potential diagnostic marker for CAD.

The miR-145 level in total peripheral blood has been found to be elevated in patients with acute myocardial infarction and correlate with the infarction size estimated by troponin-T release [36]. Since miR-145 is enriched in VSMCs, elevated miR-145 levels in AMI may reflect the vessel injury that occurs during atherosclerotic plague rupture. Consistent with this idea, upregulation of miR-145 expression is found in atherosclerotic plaques in hypertensive patients [39]. Although miR-145 levels correlated with infarction size, Meder et al. did not identify miR-145 as a good predictor for AMI using receiver operator characteristic curves [36].

Recently, Kontaraki et al. investigated the expression levels of many VSMC-modulating miRNAs including miR-143/-145 in human peripheral blood mononuclear cells in patients with essential hypertension in comparison with healthy controls. The expression levels of miR-143/-145 were decreased in hypertensive patients compared with healthy controls and the expression levels negatively correlated with 24 hr diastolic and mean blood pressure [38]. It remains to be determined whether changes in miR-143/-145 levels in human peripheral blood mononuclear cells are the results of hypertension in peripheral blood mononuclear cells or secondary to the vascular wall injury induced by hypertension. In addition, although Kontaraki et al. reported that diabetes did not affect the expression of miR-143/-145 in hypertensive patients [38], it has been reported that miR-145 was reduced in AMI patients with diabetes compared with AMI patients without diabetes [36]. Future studies with large cohorts of patients are required to confirm the role of miR-143/-145 as diagnostic markers for essential hypertension.

## 5. MiR-143/-145 as a Potential Therapeutic Targets for the Treatment of Cardiovascular Diseases

MiRNA dysregulation is linked to the development of cardiovascular diseases; restoration of dysregulated miRNAs to their normal levels can potentially reduce or even eliminate diseases, at least in animal models [80, 81]. MiRNA-based therapy has been regarded as a promising method for clinical applications in the treatment of cardiovascular diseases [13, 80]. Compared with traditional molecular targets, miRNAs have features that favor them as potential good therapeutic targets. MiRNAs may target many mRNAs that share common biological and cellular function and thus may produce a strong effect on end-point functions. In this way, targeting miRNAs may less likely cause desensitization, which occurs commonly when only one therapeutic target is aimed for in most classical drugs. In addition, targeting cell- and disease-specific expression of miRNAs may reduce off-target effects, which are severe problems for classical drugs. Therefore, identification of cell- and disease-specific miRNAs is important for successful miRNA-based therapy.

MiR-143/-145 are predominantly expressed in VSMCs and play a role in several cardiovascular diseases, such as essential hypertension [38], atherosclerosis [40], pulmonary arterial hypertension [37, 39], and coronary artery disease [35]. Targeting miR-143/-145 may be a promising therapy for these cardiovascular diseases. However, to date, no miRNA-based therapy has been developed to treat cardiovascular diseases in human clinical trials. Several animal studies have demonstrated that targeting miR-143/-145 may be a promising therapy for vascular diseases.

Given the association of aberrant expression of miR-143/-145 with the pathogenesis of many cardiovascular diseases, experimentally manipulating them to be either up or down expressed might be beneficial in the prophylaxis or treatment of these diseases. Currently, adenoviruses-mediated gene transfer system is a commonly used method which can experimentally make some target genes up or down expression* in vivo*. Through using the commercially available Adeno-X Expression Systems 2 kit, Cheng's group successfully constructed the adenoviruses which are expressing miR-145. Subsequent studies by implying this gene transfer system into rat model with carotid artery balloon injury reported that transient local adenoviral overexpression of miR-145 inhibited neointimal lesion formation in rat carotid arteries after vascular balloon injury [30], suggesting that restoration of downregulated miR-145 may be effective for the treatment of vascular diseases. Furthermore, adenoviral overexpression of smart miR-145 in which the flank sequence of miR-145 was switched with that of miR-31 results in a more inhibition on balloon injury-induced neointimal lesion formation in rat carotid arteries, compared with adenoviral overexpression of miR-145 [58], suggesting that modulation of the flank sequence may be an effective way to improve therapeutic efficacy of miRNAs. In addition, using a similar method, Elia et al. found that overexpression of either miR-143 or miR-145 reduced balloon injury-induced neointimal lesion formation in rat carotid arteries [43]. Furthermore, systemic injection of lentiviral miR-145 markedly reduced atherosclerotic volumes in aortic roots, ascending aortas, and brachiocephalic arteries of ApoE-knockout mice [59].

To date, most investigators have used virus-mediated gene transduction for overexpression of miRNAs in animals. Recently, Ohnaka et al. developed a nonviral method for transduction of miR-145 into a bypass graft, using an electroporator [82], involving the usage of an expression vector for human miR-145 (pMIW-cGFP-miR-145) which is commercially available. Their study demonstrated that MiR-145-overexpressing vein grafts inhibited neointimal lesions formation in the arterialized rabbit vein graft model [82]. This nonviral gene transfer method reduces the potential ethical issues and cytotoxicity owing to difficulties in virus handing and regulation and may be more appropriate for clinical application.

## 6. Conclusions and Perspectives

Recently, increasing evidence has shown that plasma miRNAs can be used as a potential disease biomarker [55, 76, 77]. MiR-143 and miR-145, important molecules that determine the phenotype of VSMCs, can be released into the plasma in response to vascular injury and thus may be used as potential diagnostic biomarkers for vascular diseases. Recent clinical findings have demonstrated that miR-143 and miR-145 levels in the plasma are associated with essential hypertension [38], coronary artery disease [34, 35], and AMI [36]; these findings highlight the importance of miR-143/-145 as potential biomarkers for cardiovascular diseases. However, one should keep in mind that the use of miR-143/-145 as biomarkers for cardiovascular diseases should be further investigated in comprehensive studies with a large sample size, since, to date, clinical studies have only included a small sample size. In addition, the expression levels of multiple miRNAs differ based upon the specific cardiovascular disease [78], and thus expression profiles of multiple miRNAs may be better used for diagnosis. Furthermore, it would be interesting to identify circulating miRNAs as biomarkers for predicting a response to a specific treatment in cardiovascular diseases.

MiRNAs play an essential role in the development of many cardiovascular diseases, and miRNA-based therapy has been recognized to be a promising novel therapeutic strategy for the treatment of cardiovascular diseases [13, 16, 80]. MiR-143/-145 are relatively specific for VSMCs and thus are attractive potential drug target for vascular diseases such as atherosclerosis, hypertension, and CAD. However, although the delivery of viral vectors expressing miR-143/-145 effectively inhibits neointimal lesion formation in the animal model, no clinical trials have demonstrated an effective miRNA-based therapy for the treatment of cardiovascular disease. The study with miRNA-based therapy for cardiovascular disease is just beginning. Future preclinical and clinical studies are warranted to evaluate the efficacy of miRNA-based therapy in patients with cardiovascular diseases.

## Figures and Tables

**Table 1:** Targets of miR-143/145 and associated function.

Target	Function	Reference
Ets-like gene 1 (for miR-143)	Proliferation and differentiation	[31]
KLF4 and myocardin (for miR-145)		[30, 31, 34]

MRTF-*β*	Actin modeling	[42]
Slingshot 2		[42]
KLF4 (for miR-145)		[42]
KLF5 (for miR-145)		[42]
Srgap1		[42]
Srgap2		[42]

ACE	Contractility	[29]

**Table 2:** Aberrant expression of miR-143/145-associated diseases.

Disease	miR-143/145 expression	Source of miRNAs	Reference
Essential hypertension	Down	Peripheral blood mononuclear cells	[15]
Coronary artery disease	Down	Plasma	[35]
Unstable angina	Up	Plasma	[34]
Acute myocardial infarction	Up	Peripheral total blood	[36]
pulmonary arterial hypertension	Up	Lung tissues	[37]
